# Supplementary material for: GIVE: portable genome browsers for personal websites
Source: Genome Biol. 2018 Jul 18;19:92. doi: 10.1186/s13059-018-1465-6 (PMC6050681; doi:10.1186/s13059-018-1465-6)
Supplement: Supplementary file 1 — Figure S1. Screenshot of a website hosting ENCODE datasets. Figure S2. Screenshots of GIVE data hub. Figure S3. Selection of datasets in GIVE data hub. Figure S4. HUG generated HTML code. Figure S5. Screenshot of a custom genome browser. Figure S6. Oak data structure and operations. Figure S7. Pine data structure and operations. Table S1. Summary of GIVE Toolbox. Table S2. Related to Fig. 1. Line-by-line commands and codes for creating a genome browser loaded with custom data. Table S3. Templates with real codes and complete instructions. (PDF 743 kb) [file 13059_2018_1465_MOESM1_ESM.pdf]

SUPPLEMENTARY FIGURES

Figure S1. Screenshot of a website hosting ENCODE datasets.

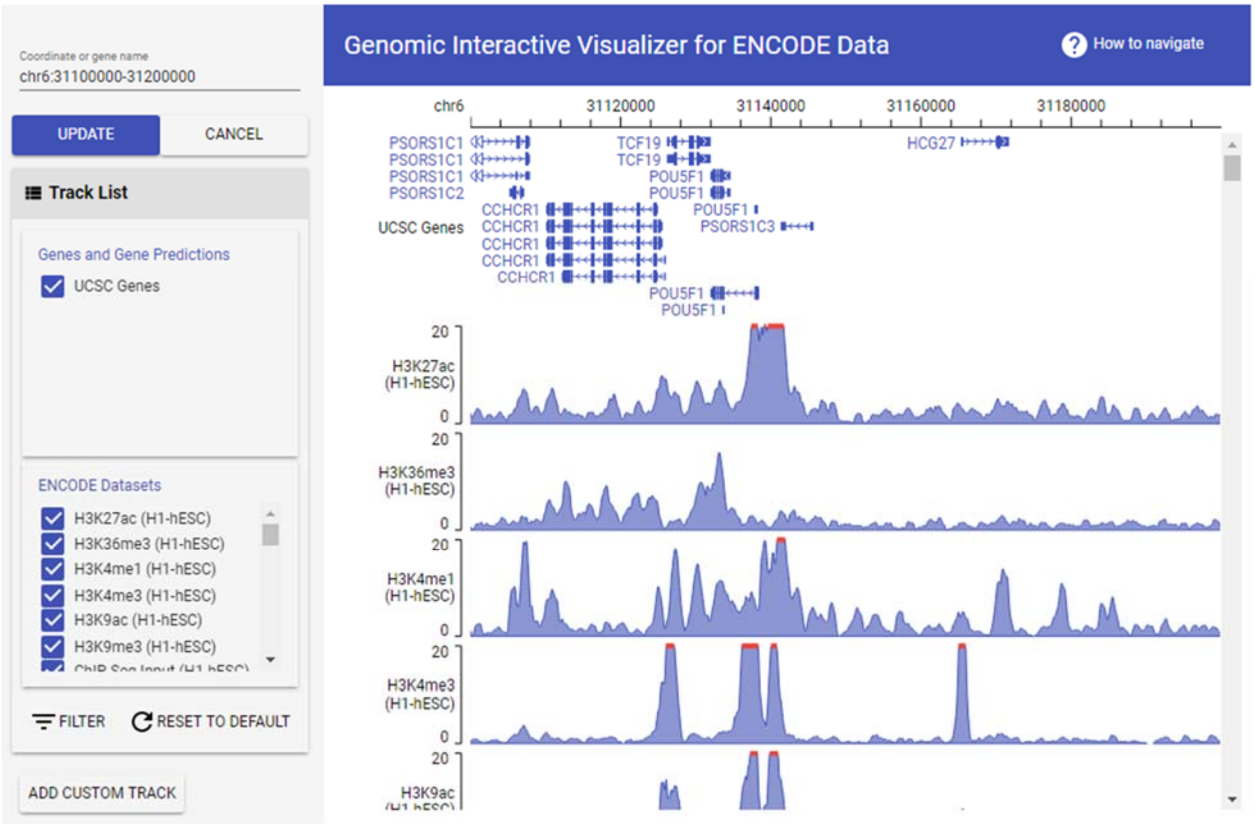

Figure S2. Screenshots of GIVE data hub. Clicking on “HTML generator mode” on the top right corner would activate GIVE HUG. Clicking on the “Filter” button will activate an interactive window (center, lower panel) for filtering datasets by data type, cell type, or lab name.

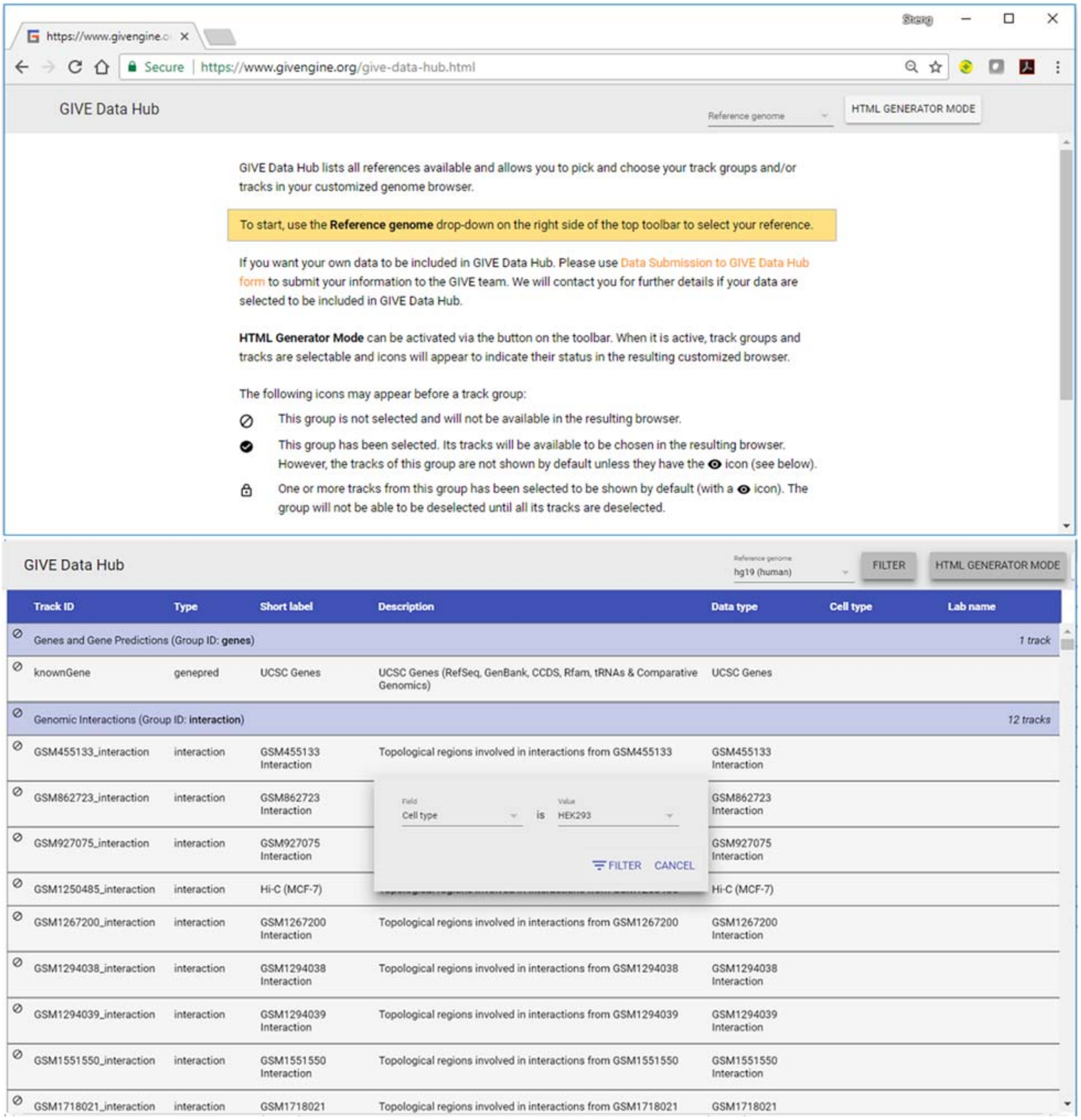

Figure S3. Selection of datasets in GIVE data hub. Clicking on a dataset will get it selected (orange) and clicking again will unselect it (yellow). Clicking on “Generate” button (upper right corner) will produce an HTML file for displaying the selected datasets. The current filter criterion “Cell type is HEK293” is displayed on the top lane. Clicking the “x” sign next to the filter criterion will remove the filter.

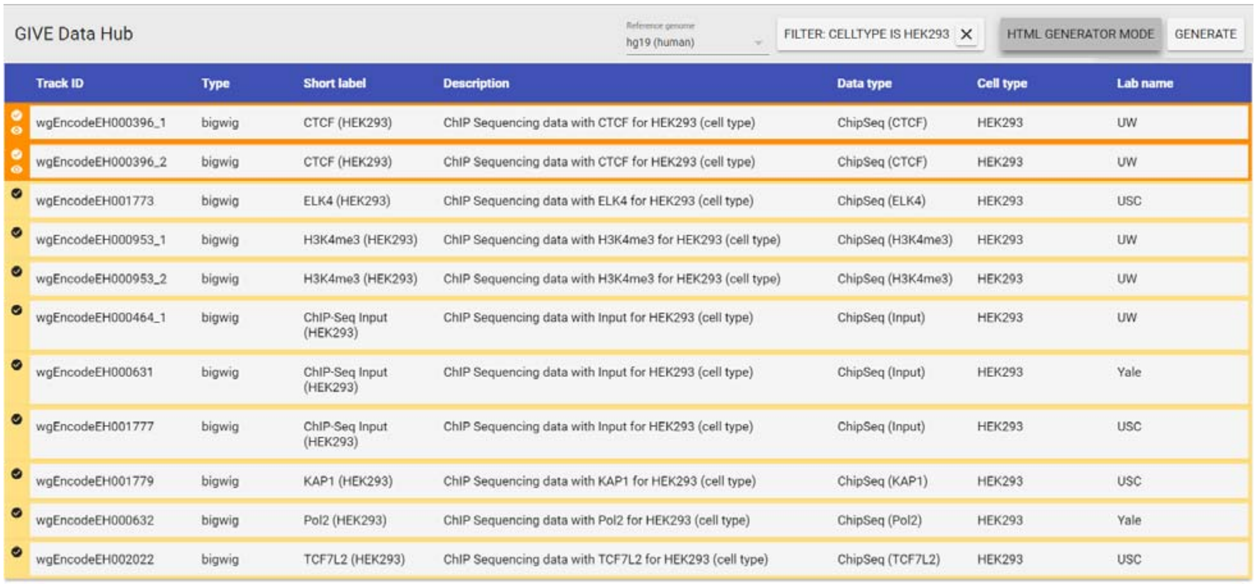

Track ID	Type	Short label	Description	Data type	Cell type	Lab name
wgEncodeEH000396_1	bigwig	CTCF (HEK293)	ChIP Sequencing data with CTCF for HEK293 (cell type)	ChipSeq (CTCF)	HEK293	UW
wgEncodeEH000396_2	bigwig	CTCF (HEK293)	ChIP Sequencing data with CTCF for HEK293 (cell type)	ChipSeq (CTCF)	HEK293	UW
wgEncodeEH001773	bigwig	ELK4 (HEK293)	ChIP Sequencing data with ELK4 for HEK293 (cell type)	ChipSeq (ELK4)	HEK293	USC
wgEncodeEH000953_1	bigwig	H3K4me3 (HEK293)	ChIP Sequencing data with H3K4me3 for HEK293 (cell type)	ChipSeq (H3K4me3)	HEK293	UW
wgEncodeEH000953_2	bigwig	H3K4me3 (HEK293)	ChIP Sequencing data with H3K4me3 for HEK293 (cell type)	ChipSeq (H3K4me3)	HEK293	UW
wgEncodeEH000464_1	bigwig	ChIP-Seq Input (HEK293)	ChIP Sequencing data with Input for HEK293 (cell type)	ChipSeq (Input)	HEK293	UW
wgEncodeEH000631	bigwig	ChIP-Seq Input (HEK293)	ChIP Sequencing data with Input for HEK293 (cell type)	ChipSeq (Input)	HEK293	Yale
wgEncodeEH001777	bigwig	ChIP-Seq Input (HEK293)	ChIP Sequencing data with Input for HEK293 (cell type)	ChipSeq (Input)	HEK293	USC
wgEncodeEH001779	bigwig	KAP1 (HEK293)	ChIP Sequencing data with KAP1 for HEK293 (cell type)	ChipSeq (KAP1)	HEK293	USC
wgEncodeEH000632	bigwig	Pol2 (HEK293)	ChIP Sequencing data with Pol2 for HEK293 (cell type)	ChipSeq (Pol2)	HEK293	Yale
wgEncodeEH002022	bigwig	TCF7L2 (HEK293)	ChIP Sequencing data with TCF7L2 for HEK293 (cell type)	ChipSeq (TCF7L2)	HEK293	USC

Figure S4. HUG generated HTML code. Top row summarizes user selected datasets, including reference genome, data group and short descriptions of selected datasets. The generated HTML code is shown in the lower half of this window. Users can use the interactive features in the middle portion of this window to modify display parameters. Clicking “Update code” button will refresh the lower half with a regenerated code incorporating the new parameters.

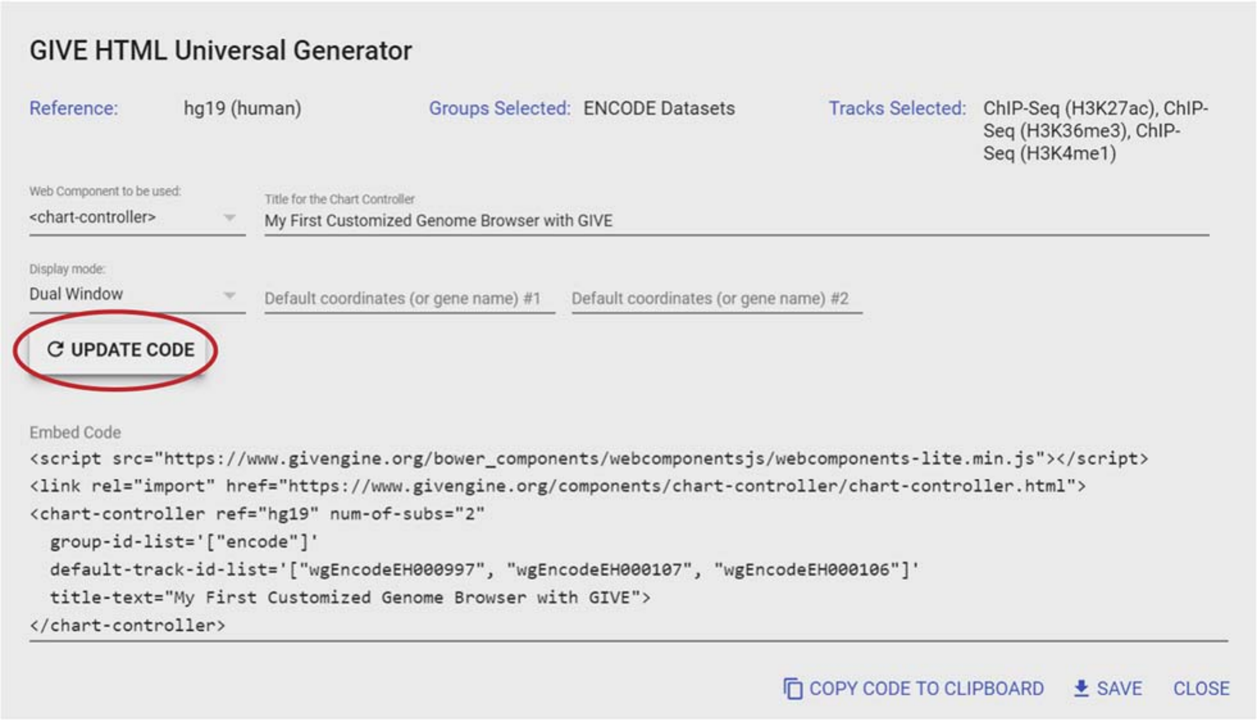

Figure S5. Screenshot of a custom genome browser. (A) Text box and “update” button for changing genomic coordinates. (B) Check boxes to choose datasets for retrieval and display. (C) Genome coordinates and genes. Dragging with mouse will shift genome coordinates. Rolling the mouse wheel will zoom in or out.

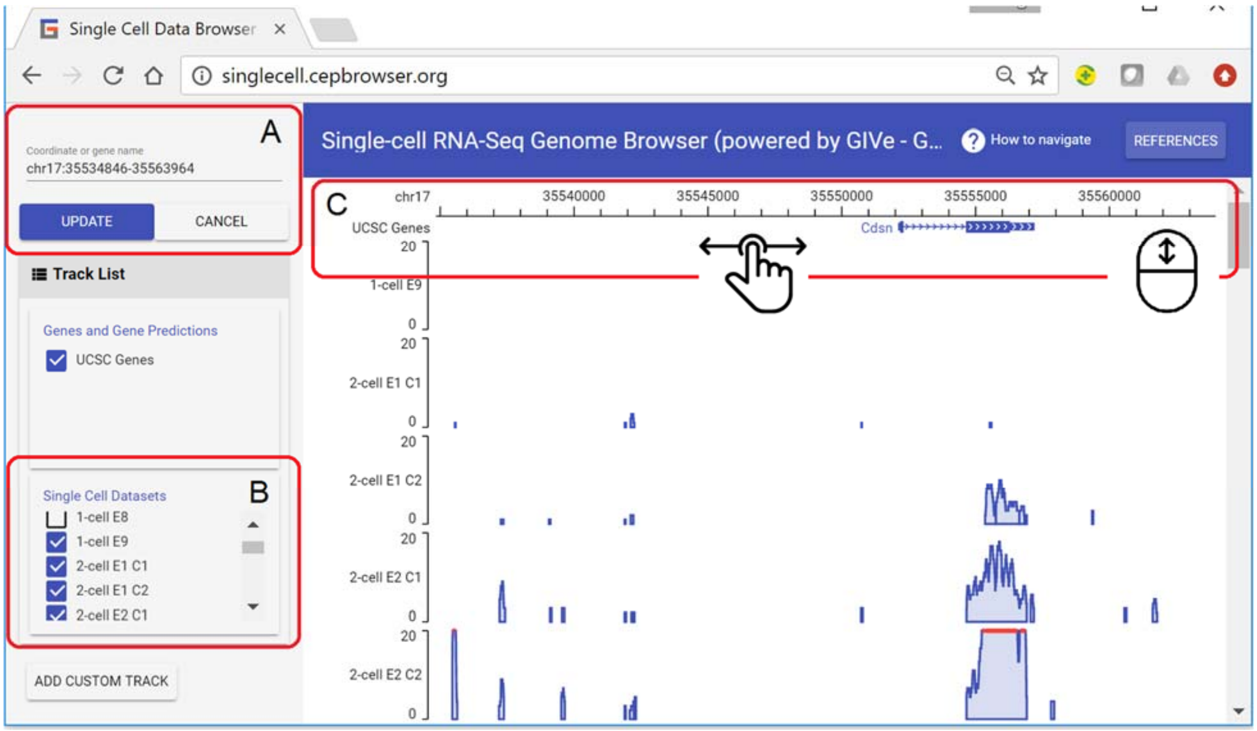

Figure S6. Oak data structure and operations.

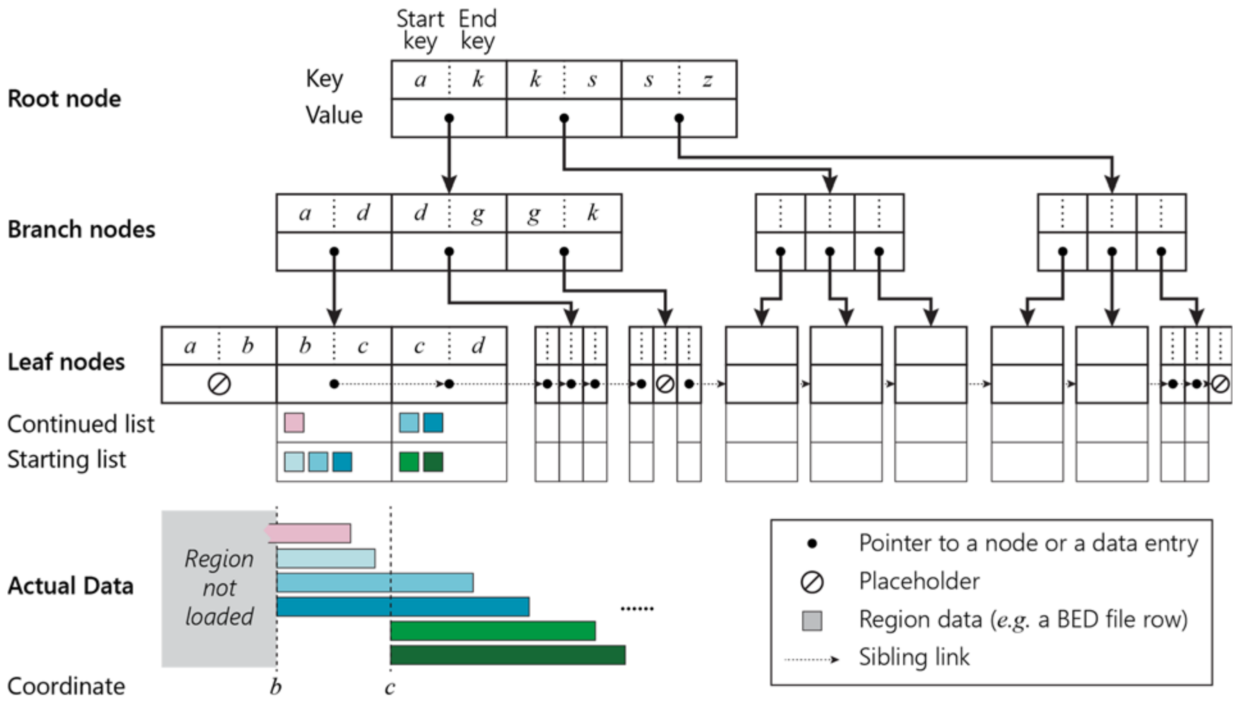

Figure S7. Pine data structure and operations.

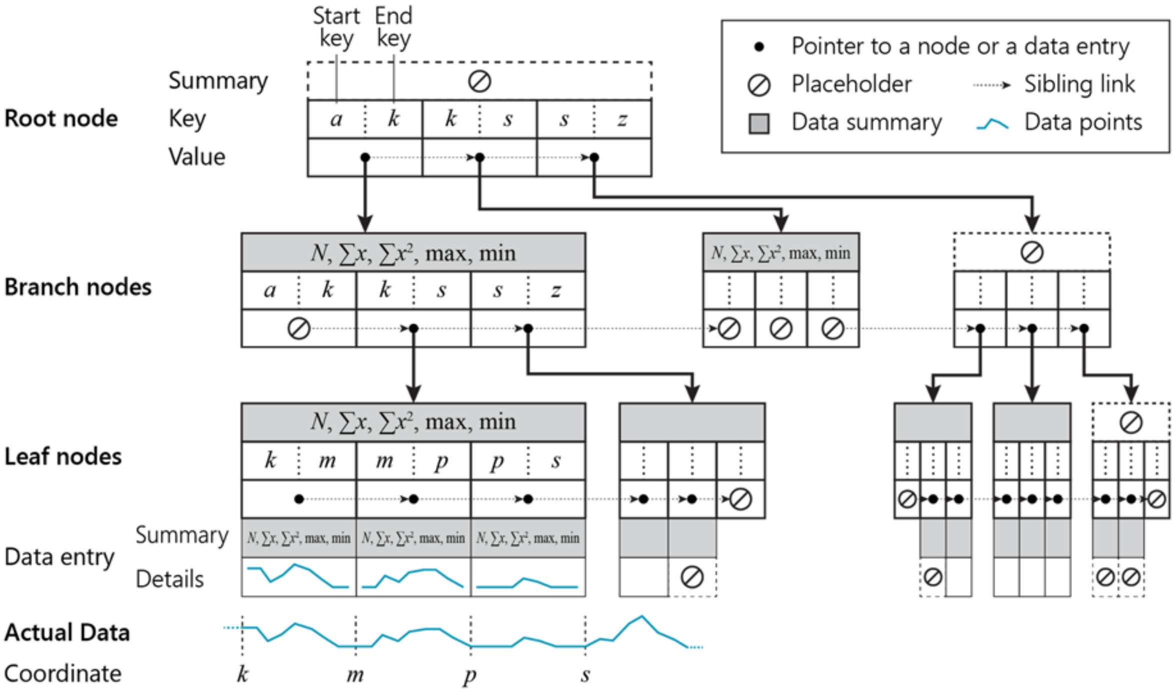

SUPPLEMENTARY TABLES

Table S1. Summary of GIVE Toolbox. GIVE Toolbox is a set of command line commands (Command) that automates databased operations (Operations).

Goal	Command	Operations
**Initialize a reference genome**	initial_ref.sh	Create a database of the reference genome
		Create a table for the chromosome sizes
		Import data to the chromosome sizes table
		Register the reference genome database
		Create a table for track group registration
		Create a table for track registration
**Create a data track**	add_trackGroup.sh	Register the track group that the track belongs to
	(choose based on data type)<br>add_geneAnnot.sh<br>add_track_bed.sh<br>add_track_bigWig.sh<br>add_track_interaction.sh	Create a table for the data track
		Import data to the data track table
		Register and add annotation (metadata) of the data track
**List existing data**	list_tracks.sh	Check registration tables of reference genome, track groups and data tracks one by one
**Remove data**	remove_data.sh	Drop the data track table
		Delete the info in related registration table

Table S2. Related to Figure 1. Line-by-line commands and codes for creating a genome browser loaded with custom data. The first three steps (Step column) were automated as command line commands (Command/HTML tag) in GIVE-Toolbox (Component). The final step utilizes an HTML tag provided in the HTML tag library (Component).

Step	Line by line command or code	Component	Command/HTML tag
**Initiate a reference genome**	Create data files for the reference genome	GIVE-Toolbox	initial_ref.sh
**Add gene annotations**	Create a track group for gene annotations		add_trackGroup.sh
	Add gene annotation file		add_geneAnnot.sh
**Load custom data**	Create a track group for custom data tracks		add_trackGroup.sh
	Load a custom data file		add_track_bigWig.sh
	Repeat the last step to add other data files		add_track_bigWig.sh
**Display data**	Insert an HTML tag to display the data	HTML tag library	<chart-controller>

Table S3. Templates with real codes and complete instructions. All these demos are accessible via the hyperlinks in this table and available in “GIVE Tutorial” from GIVE’s homepage (www.givengine.org).

Number	Title
1	Start from a 2-minute example
2	Build a genome browser with GIVE data hub
3	Local deployment of GIVE with GIVE-Docker
4	Use GIVE-Toolbox to manage custom data
5	Demo of long-range promoter-enhancer interactions with capture Hi-C
6	Demo with ChIA-PET derived chromatin interactions
7	Demo with superimposed wiggle and genomic interaction tracks
